# Behavioral recovery from traumatic brain injury after membrane reconstruction using polyethylene glycol

**DOI:** 10.1186/1754-1611-2-9

**Published:** 2008-06-27

**Authors:** Andrew O Koob, Julia M Colby, Richard B Borgens

**Affiliations:** 1 Center for Paralysis Research, Program in Neuroscience, Purdue University, West Lafayette, IN 47907, USA; 2 Center for Paralysis Research, Department of Basic Medical Sciences, Purdue University, West Lafayette, IN 47907, USA; 3 Center for Paralysis Research, Department of Basic Medical Sciences, Department of Biomedical Engineering, Weldon School of Biomedical Engineering, School of Veterinary Medicine, Purdue University, West Lafayette, IN 47907, USA; 4 Stein Institute for Research on Aging, University of California, San Diego, La Jolla, CA 92093, USA

## Abstract

Polyethylene glycol (PEG; 2000 MW, 30% by volume) has been shown to mechanically repair damaged cellular membranes and reduce secondary axotomy after traumatic brain and spinal cord injury (TBI and SCI respectively). This repair is achieved following spontaneous reassembly of cell membranes made possible by the action of targeted hydrophilic polymers which first seal the compromised portion of the plasmalemma, and secondarily, allow the lipidic core of the compromised membranes to resolve into each other. Here we compared PEG-treated to untreated rats using a computer-managed open-field behavioral test subsequent to a standardized brain injury. Animals were evaluated after a 2-, 4-, and 6-hour delay in treatment after TBI. Treated animals receive a single subcutaneous injection of PEG. When treated within 2 hours of the injury, injured PEG-treated rats showed statistically significant improvement in their exploratory behavior recorded in the activity box when compared to untreated but brain-injured controls. A delay of 4 hours reduced this level of achievement, but a statistically significant improvement due to PEG injection was still clearly evident in most outcome measures compared at the various evaluation times. A further delay of 2 more hours, however, eradicated the beneficial effects of PEG injection as revealed using this behavioral assessment. Thus, there appears to be a critical window of time in which PEG administration after TBI can provide neuroprotection resulting in an enhanced functional recovery. As is often seen in clinically applied acute treatments for trauma, the earlier the intervention can be applied, the better the outcome.

## Background

Traumatic brain injury (TBI) is a pernicious event that destroys many lives. Currently, there are no effective pharmacological treatments for TBI [1]. Treatments once thought promising such as the use of corticosteriods have been determined not to have any neuroprotective qualities [2,3].

PEG has been used subsequent to a standardized TBI in the rat to reduce cellular damage in brain in various regions, particularly the white matter of the corpus callosum [4]. Additionally, immunohistochemical staining for β-Amyloid Precursor Protein, a protein that aggregates in the axon terminal associated with progressive secondary axotomy, has been significantly reduced by PEG treatment after injury [5]. The salutary effects of PEG have likewise been seen in the injured spinal cord. The mechanical interaction of the polymer with only damaged membranes leads to rapid (minutes to hours) improvements in physiological function, permanent repair of the membrane damage, inhibition of free radical production, and reduction in the size of progressive cavitation [6-8]. These actions result in an improved behavioral recovery after laboratory spinal cord injury (SCI) in guinea pigs and rats, and in clinical cases of paraplegia in dogs [9].

PEG's membrane sealing and reassembly properties were originally utilized for vesicle fusion and hybridoma formation [10,9,11]. PEG molecular weights < 4 kD are sufficient to produce cell and axon fusion [12,7], recovery of conduction in white matter tracts after crush injury [13], and a reduction in the destruction of axons after spinal cord crush injury [6,14].

The studies describing the neuroprotective properties of PEG after brain injury persuaded us to test the effect of PEG injection on rat behavior after TBI. In the rat, the same Impact Acceleration injury model as used in the present investigations produced significant behavioral impairment [15,1]. Brain-injured animals are impaired in "open-field" evaluations, the "rotorod" behavioral evaluation, and on the beam walk [16,15]. Open-field exploratory behavior is the most ideal preliminary test to apply to injured animals because this evaluation does not require "blinding" of the investigator, as this person is not involved with the taking, or generating of the data. Furthermore, the natural exploratory behavior of the rat provides the baseline function which also does not require training but does require cognitive, motivational, and motor performance capabilities of the study animals.

## Methods

### Animals

All rats studied were 400–450 gram, adult, Sprague-Dawley rats (Hilltop Lab Animal Inc. Indianapolis, IN, USA). They were fed (Purina Rat Chow #5001) and allowed water ad libidum. The animals were housed in a climate-controlled facility with a 12:12 hour light:dark cycle. All animal procedures were submitted and approved by the Purdue University Animal Care and Use Committee, with strict attention to university, state, and federal guidelines for the use of animals in research.

### Surgical Procedures

Rats were anesthetized with 4% isoflurane in 99% oxygen (Vetamac, Inc., Rossville, IN, USA, VAD compact anesthetic machine with vaporizer) in a laboratory-fabricated gas chamber. They were intubated endotracheally (Intramedic polyethylene tubing, I.D. 1.57 mm, O.D. 2.08 mm, Fisher Scientific, Hampton, NH, USA) and ventilated with 1.5–2.5% isoflurane in 99% oxygen on a Harvard Apparatus small animal ventilator (Holliston, MA, USA). Body temperature was maintained with a Harvard Apparatus homeothermic blanket control unit. A midline scalp incision was performed, and the periosteum removed to expose the skull. A metal disk, 10 mm in diameter, 3 mm thick, was firmly attached with Loctite super glue gel and dental acrylic (Henry Schein, Melville, NY, USA) to the skull between the lambda and bregma sutures. A 450-gram brass weight was dropped from a height of two meters onto the disc to induce a severe impact/acceleration injury using this injury model [4,5,17]. All anesthetized animals received a 0.075 mg/kg intra-muscular injection of Buprenex and were weaned off the ventilator within an hour of injury.

### General conduct of the study

After laboratory induced TBI using the Marmarou model [4,5,17] a delay in PEG administration of 2, 4, and 6 hours was tested. (A pilot experiment where PEG injection was used within the first 15 minutes of the head injury will be mentioned only briefly, and complete data is not given).

Nineteen animals of the total of 47 animals used in studies of the 2–6 hour delay were eliminated from the study due to death, skull fracture, or euthanization due to progressive medical problems, an outcome due to using the most severe form of impact. Of the balance, five rats underwent a "sham injury" procedure. These received the same procedures as the injured groups, however the weight was allowed to drop two to three inches from the subject's head. These will be described in the text as "sham injured" or "uninjured" animals. Six "control" rats received a subcutaneous injection of sterile saline, while the experimental groups all received subcutaneous injections of PEG at three time point post injury: six animals at 2 hours, and 8 animals each after a 4-hour, and 6-hour delay in PEG administration. After behavioral testing, animals were sacrificed with an overdose of sodium pentobarbital.

### Open-field testing

We direct the interested reader to significant details of the "activity chamber" construction, the acquisition, management, and validation of data in Koob et al 2006. A 30-minute period of behavioral evaluation in the activity box was used in these investigations. After experimental TBI, a subcutaneous injection of PEG was given at 2, 4, and 6 hours later. Behavioral evaluations were made at 24 hours, 4 days, and 7 days post-injury.

**Figure 1:**
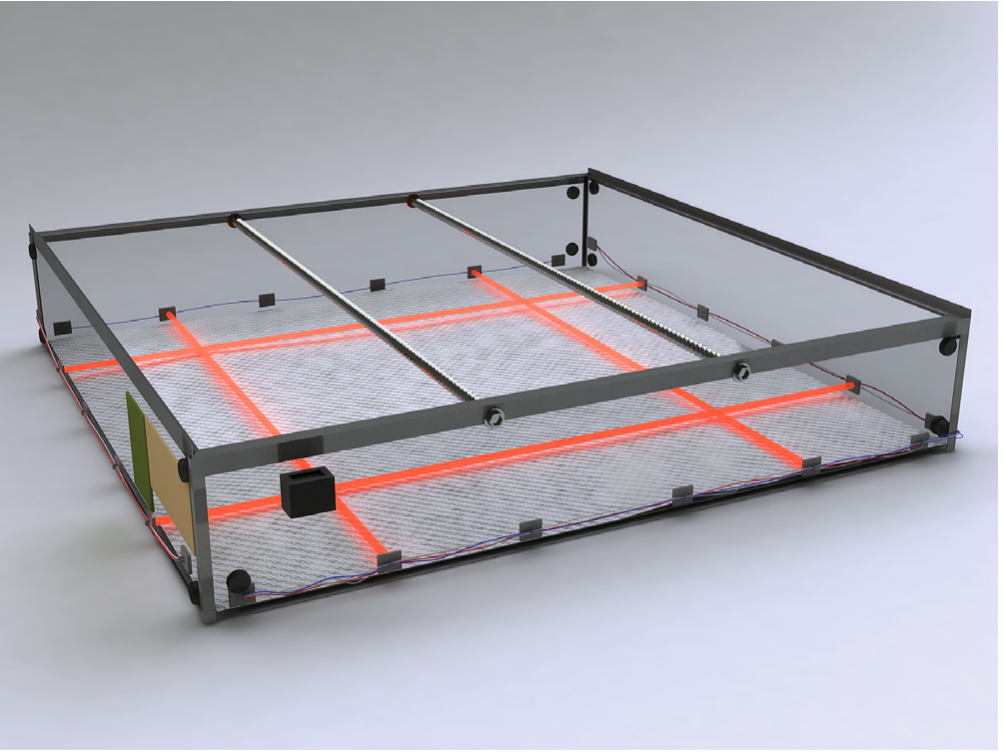
**"Open field" evaluation using the activity chamber**. In the graphic, infrared beams are rendered as red, and these four beams for each axis are equidistant (20 cm) apart around the perimeter of the chamber (though in the drawing only two are shown in each axis for simplicity). The box is 100 × 100 × 20 cm. The beams are 4.5 cm high relative to the bottom of the chamber. The rat is placed in the middle of the chamber at the beginning of the test and usually allowed to explore the area for 30 minutes. Food was placed over the center of the box on top of a grid screen (not shown). A totalizer/counter registered the number of beams broken during the period of study. A computer with laboratory designed software (see Koob and Cirillo, 2006 for details and procurement) registered which individual beam was broken to determine where the rat was in space and time.

Animals were placed in a 100 cm × 100 cm × 20 cm Plexiglas activity chamber in a darkened room (the rat's natural exploratory is most elevated during nocturnal periods). Inserted at equidistant points around the perimeter of the box, 4.5 cm off the base, four infrared beams in the *X *and *Y*-axis provided data as to the subject's location at any given point (Fig 1). The smallest sector that the rat can cross is thus 20 cm × 20 cm. Movements inside the 20 × 20 cm region could be important, however this spacing was chosen to allow an entire animal to fit inside. In this initial investigation, we were interested in the rat's ability to walk (while exploring) after TBI, as the injury is detrimental to motor and cognitive behavior, but especially motor behavior in this injury model [15].

Software designed especially for use with the activity box interpreted the generated series of data points to determine the animal's location in space and time over the course of the experiment (for details and to obtain software see [18]).

**Figure 2:**
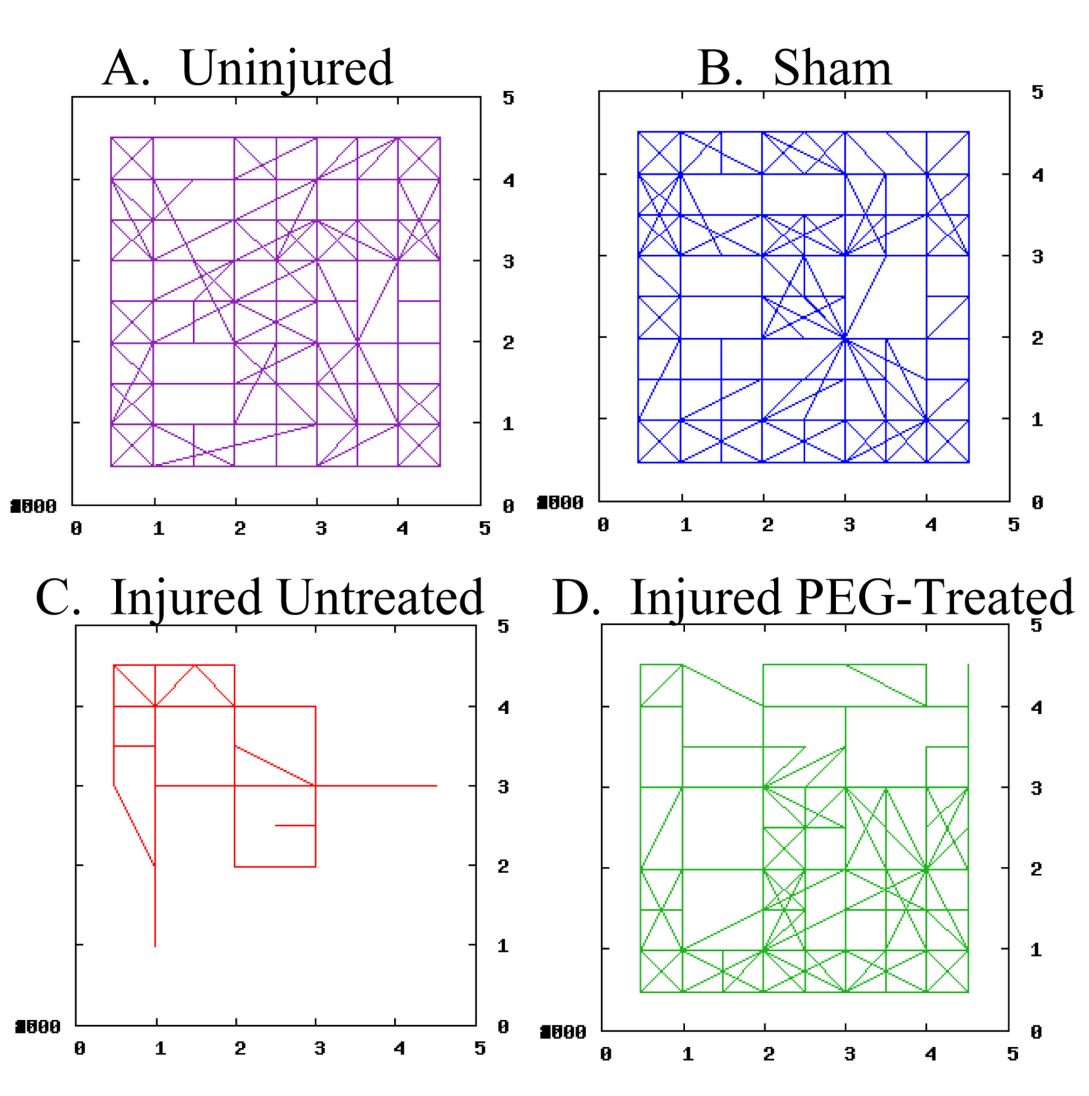
**Rat open field exploration: 2-D representation**. A 2-D representation of rat open field movements as plotted from the pilot experiments. One hour (3600 seconds) in the box was used here for demonstration purposes but not in continuing studies. The x and y axis (1–4) corresponded to the beams crossing the chamber. Using novel computer management of this data (see methods), the rat's place in space and time was determined. In A), an uninjured animal explores much of the area over the course of an hour. B) A sham injured animal also explores most of the area. In C), an injured untreated animal only explores one corner of the box. And in D) an injured PEG-treated animal covers much more area compared to C.

**Figure 3:**
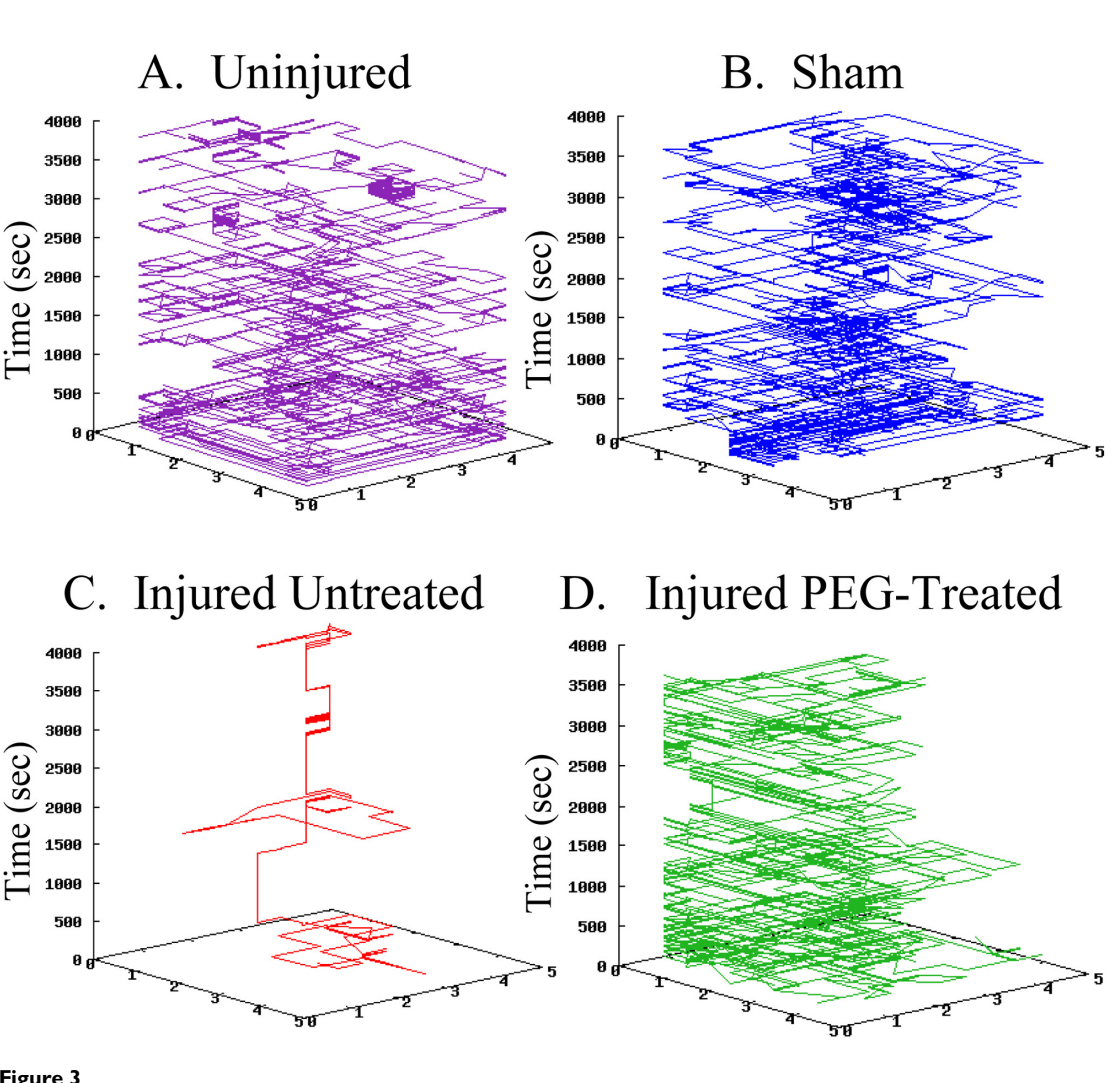
**Rat open field movement: 3D representation**. The same data from the previous figure are replotted as a three-dimensional graph(3D); z-axis in seconds (3600 sec). The x and y axis (1–4) correspond to the beams crossing the chamber. In A), an uninjured animal explores virtually the entire space. InB) a sham injured animal also explores much of the entire chamber. In C), an injured untreated animal does not move much and prefers to sit, stationary (vertical lines), thus exploring little. In D), an injured PEG-treated animal explores significantly more space than an injured untreated animal as revealed by these 3-D graphs.

Coordinates of the rat's position in space over time can be converted using a spreadsheet (Microsoft Excel, Microsoft, Seattle, WA, USA) for statistical measurements and the generation of 2- and 3-dimensional graphs (Fig 2 and 3). Here, four outcome measures were derived from these data;:percentage of the area explored, distance traveled, average speed while exploring, and the percent time spent exploring.

#### 1. Distance traveled

The distance each subject traveled over the course of data collection was determined by placing the raw data coordinates of the rat's position into a spreadsheet where each beam on each axis is assigned an integer, 1–4;*X*_1 _and *Y*_1 _are the previous coordinates and *X*_2 _and *Y*_2 _are the following coordinates. The equation *d *= √ [(*X*_1_-*X*_2_)^2 ^+ (*Y*_1_-*Y*_2_)^2^] determines distance traveled at each 200 msec interval of time. Knowing that the distance between each beam is 20 cm, the resulting sum of distance traveled for all time points over the course of the session is multiplied by 0.2 to give a distance in meters.

#### 2. Average rat speed (m/sec)

Average speed was calculated by aforementioned distance measurement in meters over the time of the session in seconds.

#### 3. Percent time exploring

In-house developed computer software collected the rat's coordinates at 200–250 msec intervals, computed the total number of coordinate pairs recorded and number of changes in coordinates throughout the exploration session. Positional coordinate changes indicate movement, thus the number of coordinate changes divided by the total data points recorded equaled the percentage of time spent exploring. As used here, "Percent Time Exploring" reflects not simply exploratory behavior but how quickly and actively the rat moves through the activity box.

#### 4. Percent Area Explored

To determine the amount of area explored, the coordinates of the rat's position were placed into a spreadsheet file and graphed. If at any given point in time a beam was broken in the *X *or *Y-*axis, the rat's position could be determined. If beams were not broken, the subject's position could be determined based on the last beam broken and next beam broken. Therefore, the position of the rat in space and time was either determined to be in front of a beam or between two beams, therefore lending a clear nine point by nine point grid of potentially explored location points. Percent area explored was calculated from this 81 point-location grid.

## Results

### Overview

An "immediate" intravenous injection of PEG (a proof of concept pilot study: injection within 15 min of injury; Fig 2, 3; complete data not shown) and the two-hour and 4-hour delay in subcutaneous injection produced similar recoveries of behavioral performance in the activity box. After a 2-hour delay in PEG injection, response to the subcutaneous injection of the polymer occurred in 3 of the 4 outcome measures, statistically significantly improved over controls. Though a trend towards significance was observed in measurement of the "percentage area explored", at this sample size it did not reach significance. There was no evidence of enhanced performance when the delay of PEG injection was extended to 6 hours.

### The Delayed Application of PEG

#### Total distance traveled

**Figure 4:**
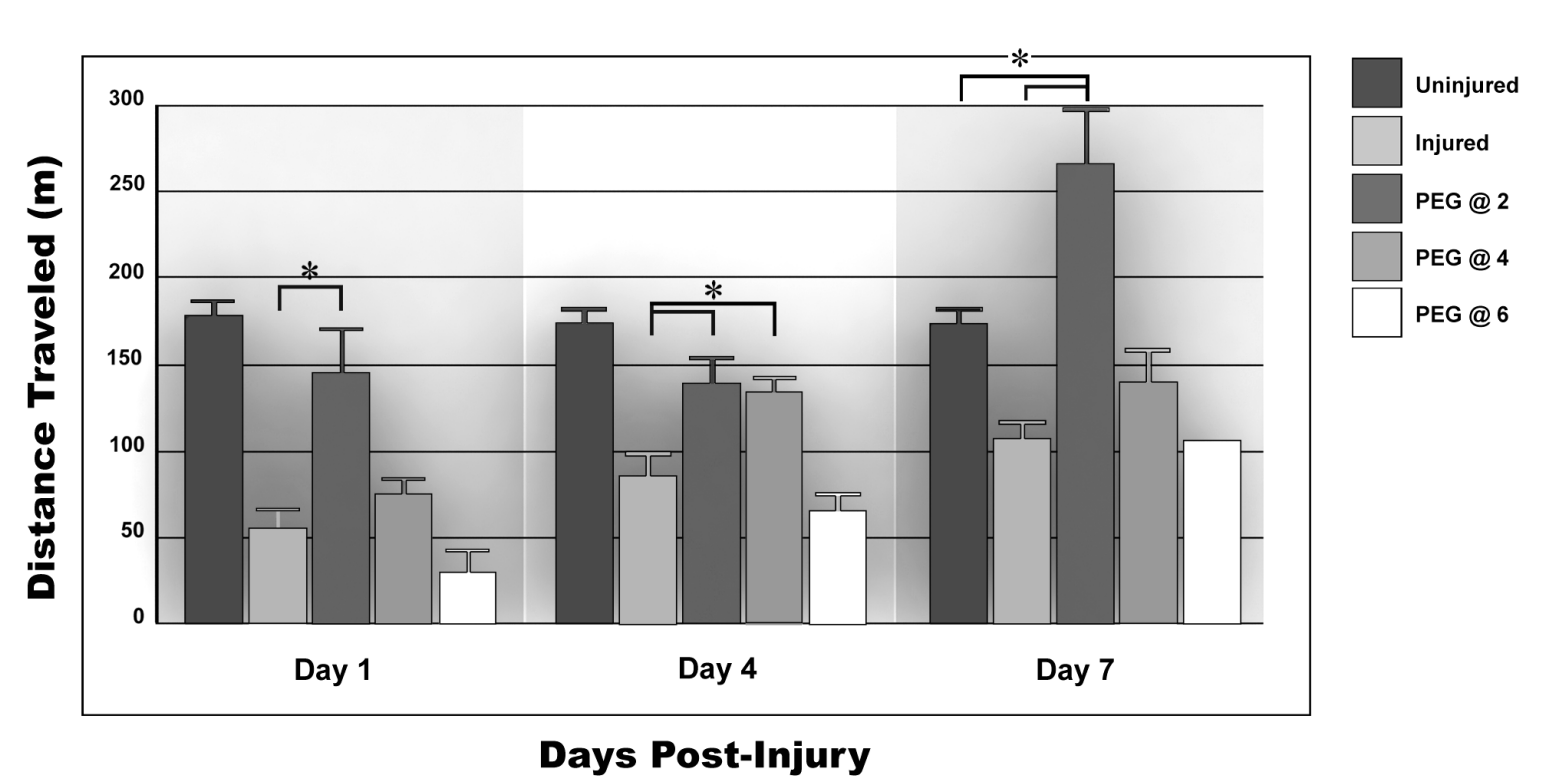
**Distance traveled**. The mean total distance traveled by rats for their time in the activity chamber. Note the marked decline in distance traveled occurring in response to TBI. The improvement produced by the 2-hour delay administration of PEG was statistically significant at 1 day, 4 days, and 1 week post-injury (P ≤ 0.05). The 4-hour delay improvement reached significance at 4 days but showed only a trend towards significance by 1 week postinjury. The 6 hour delayed administration of PEG eliminated any trend towards recovery. Note that in this caseand for all subsequent figures, the precipitous loss in performance produced by TBI was statistically significant. Brackets and asterisks are not used to denote this fact in order to simplify reading of the graph.

When the total distance traveled was compared at all time points after treatment, the 2-hour delay group was significantly improved (P ≤ 0.01). A trend towards improvement, but statistically insignificant, was noted at the end of the first day post-injury following the 4-hour delayed injection. By 4 days post-injury however, this trend in improvement had become statistically significant (P ≤ 0.05; Figure 4). While the trend towards significance in the 4-hour delay group was still apparent at 7 days post-injury, it did not reach significance at this time.

Rats that received a 6-hour delayed injection performed worse than even controls in all measurements described here and below, though this difference was not statistically significant with the exception of data obtained on day 4. There was also an increase in the distance traveled and average speed (see below) after a 2-hour injection of PEG relative to uninjured animals. The absence of an error bar indicates an SEM that cannot be distinguished from the mean in the bar graph.

#### Average speed attained while exploring

**Figure 5:**
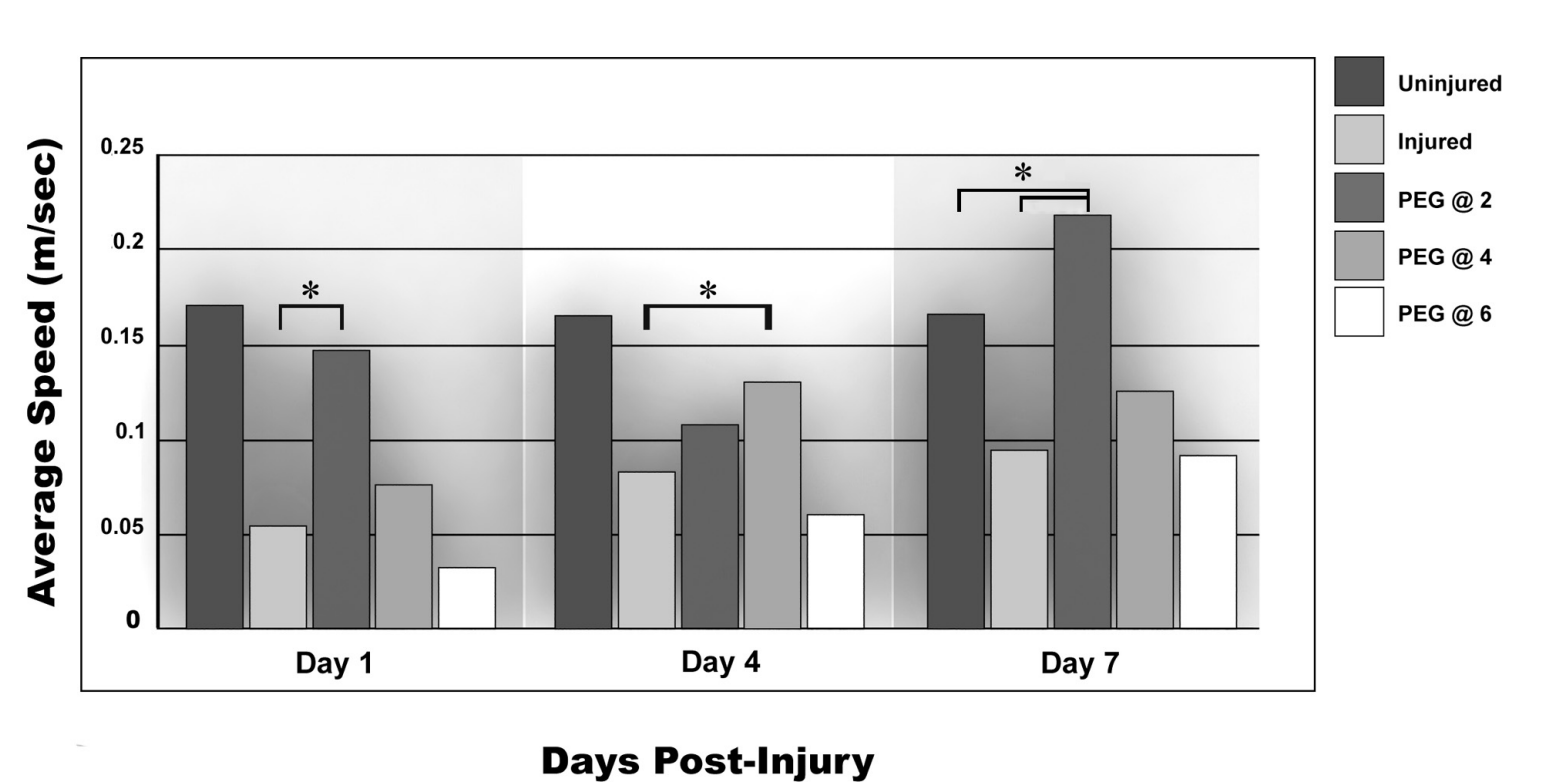
**Average speed attained**. Similar to the data in Figure 4, a striking increase in speed was achieved by the 2-hour delayed PEG administration at 24 hours and 1 week post-injury (P ≤ 0.01); and by the 4-hour delayed PEG administration at day 4 post-injury. The 4-hour delay improved this outcome measure at 7 days post-injury, but the increase was not statistically significant. The 6-hour delay failed to produce any improvement. Note that these data were especially tightly grouped, and SEMs were not drawn onto the bar graph as they were too close to the mean to be easily discerned.

There was a modest trend for improvement in the average speed attained after PEG injection compared to injured yet untreated animals. This increase only reached significance, however, for PEG-injected animals when administered at 2 hours (day 1 and 7), and for the 4-hour delayed injection at day 4 (Figure 5). Similar to above, the 2-hour delay in PEG injection led to an apparent increase in speed compared to untreated animals while further delay in treatment did not reveal this gain in performance.

#### Percentage of the time in the activity box spent exploring

**Figure 6:**
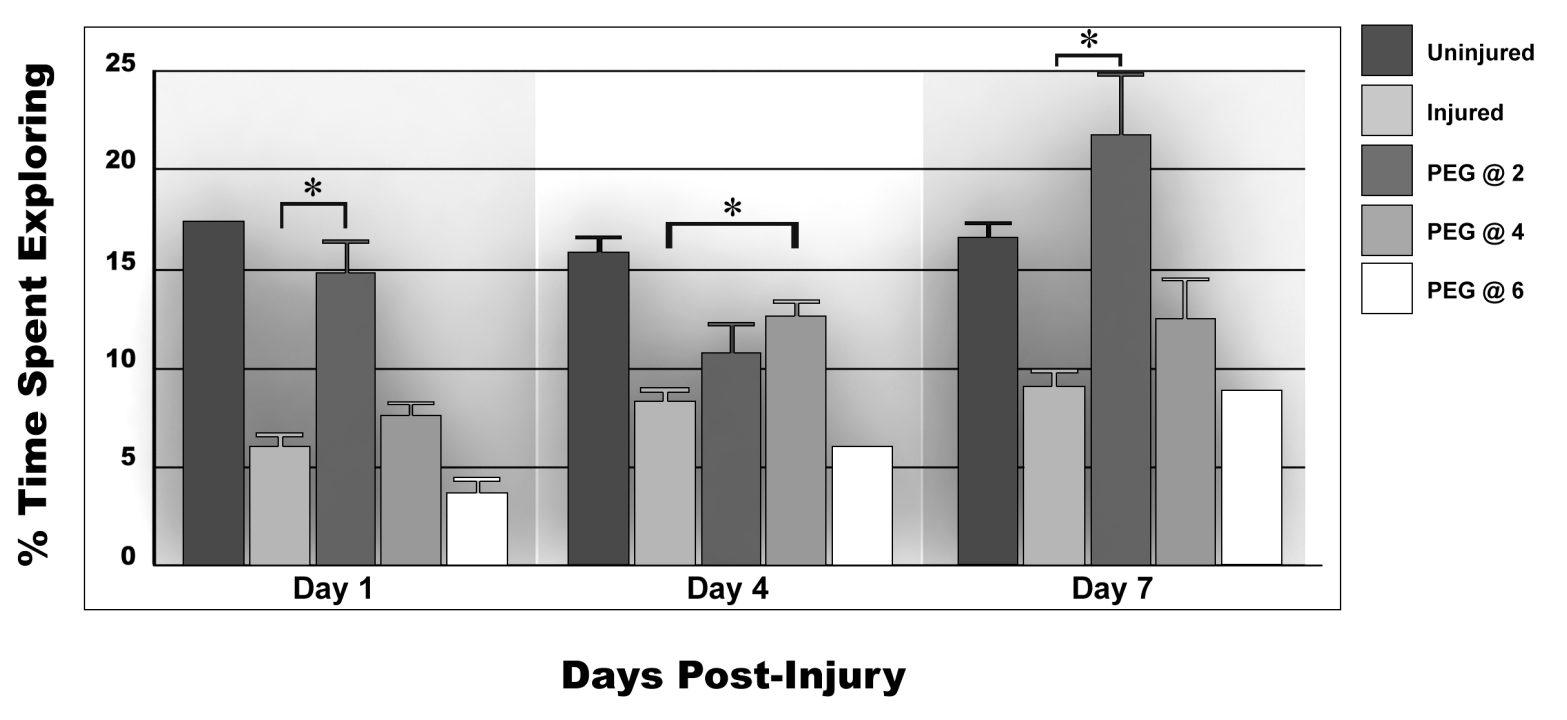
**The Percentage of time spent exploring the activity chamber**. As seen in previous outcome measures, the 2-hour delay showed a significant improvement over untreated animals at 1 day and 7 days post-injury (P ≤ 0.01), though not reaching significance at 4 days post-injury. The 4-hour delay also showed a trend in improvement at these time points but reached significance at only the 4 days post-injury evaluation (P ≤ 0.05). A 6-hour delay in the administration of PEG failed to produce any improvement in this outcome measure.

Delaying PEG administration by 2 hours produced a statistically significant increase in the percent time spent exploring compared to injured yet untreated animals on day 1 (for the 2-hour group); day 4 (for the 4 hour group) and at day 7 for both the 2- and 4-hour delayed injection (Fig 6; P ≤ 0.05). There was no improvement in performance observed after a 6 hour delay in PEG administration.

#### Percent area explored

**Figure 7:**
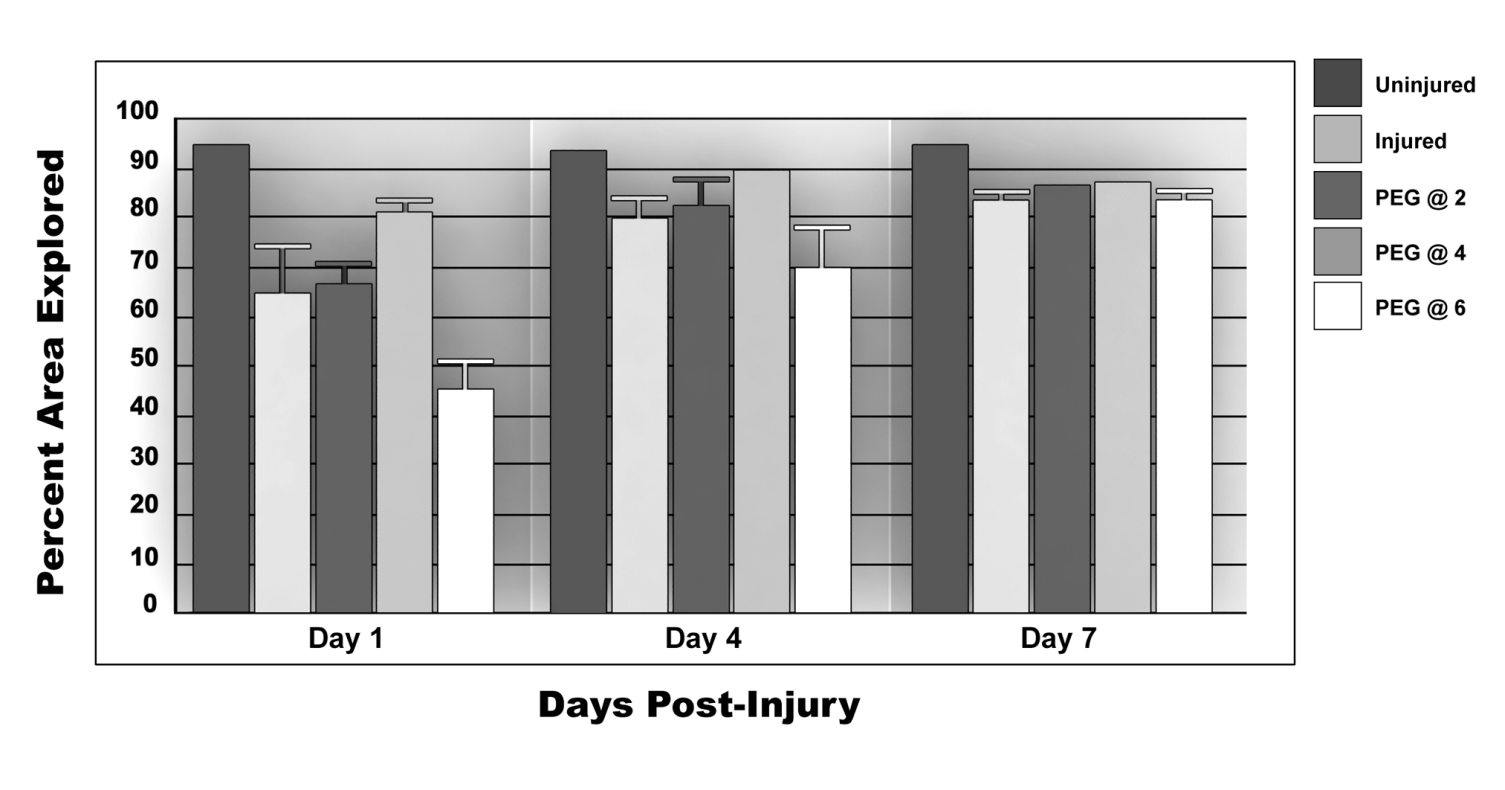
**The percentage of the area explored by rats**. When evaluating the area of the activity chamber explored by each group, it was found that all rats explored their extent of their confines to an equal degree. There was no statistical difference between any of the comparators. The absence of a SEM error bar indicates these data were to close to the mean to be plotted.

Though there was a weak trend in improvement associated with the 2- and 4-hour delayed administration of PEG, none of these improvements were statistically improved relative to Control rats (Fig 7). It is also likely that in this outcome measure, a spontaneous and progressive recovery in untreated animals also negatively impacted these data reaching significance. Overall, it is important to note that the behavioral improvements recorded did not disappear with longer times of observation (out to one week for all studies).

## Discussion

These data suggest that a delay in PEG administration of 2 to 4 hours after TBI does little to diminish its neuroprotective capability. A delay of 6 hours between injury and treatment on the other hand eliminates this protective capability as assayed by the open field test. Moreover, in nearly all evaluations, the 6-hour delay appeared to worsen the animal's outcome from TBI when compared to untreated animals. Though this observation was not statistically significant in any but one of the comparisons, the trend is undeniable. In these latter comparisons, there was no reverse trend observed at any time point for any PEG injected animal. We emphasize that this interpretation should not be extended to other behavioral examinations without investigation, though it confirms the generally held notion that a delay of many hours in treating acute CNS injury reduces or nullifies the benefits of the therapy. For example, a delay of 8 hours or more in the administration of Methylprednisilone eliminates its neuroprotective effect in treating acute spinal cord injury. PEG injections at a therapeutic dose have been given to both *uninjured *rats and to dogs [19]. In some cases, the reaction appears to produce a mild lethargy or increased "nervousness" (we note the increase in activity after the 2-hour injections in some outcome measures, see Figs. 4 and 5). However, the degree to which this might impact behavior after Brain or Spinal Cord Injury is extremely difficult to assess and thus we have emphasized only the response to PEG injections relative to controls in injured animals. Applying "Occam's Razor," we conclude that it was the 6-hour long delay alone that was responsible for the lack of behavioral recovery from TBI.

Injections similar to those used here are correlated to a significant sparing of brain parenchyma, and a sealing of neuronal membranes when PEG was administered in the acute phase of the injury [4]. Sealing of both neurons and axons in response to PEG was assayed by the uptake of extracellular markers such as Horseradish peroxidase (HRP) and Ethylene Bromide that gain entrance to the cytoplasm only through the collapse of neuronal membrane properties[4]. We also evaluated the presence of β-Amyloid Precursor Protein in injured axons as a marker for secondary axotomy [18]. In all cases, significant sparing and rescue of nerves and their processes occurred in response to acute injection of the polymer.

### Considering the two dominant forms of CNS Neurotrauma

These results stand in contrast to our published findings in SCI. In SCI, a similar program of development was used; initially even an immediate and topical PEG administration to the exposed spinal lesion was employed [7,13]. Later the administration type evolved to subcutaneously administered PEG [20], though IV was still favored by clinicians in a veterinary clinical trial administering PEG to paraplegic dogs [19]. In the guinea pig SCI model, PEG could be withheld for up to 8 hours without a diminution of its potency and, in the aforementioned clinical cases, up to 72 hours [19].

It is both instructive and convenient to consider the anatomical basis for the behavioral deficit following TBI and SCI, as they are indeed significantly different, providing some insight into the difference in response to polymer injection in these two regions of the CNS. Most importantly, white matter is particularly resistant to many of the factors associated with secondary injury processes in the CNS such as oxygen and glucose deprivation [21]. The behavioral deficits that result from spinal cord injury are nearly all based on the physiological discontinuity after damage to the long tracts of white matter. It is useful to consider the catastrophic behavioral loss after spinal cord injury as an electrophysiologically based outcome where the failure in nerve impulse conduction between brain and body is responsible for the vast majority of functional deficits. Said another way, after SCI, damage to gray matter plays a minor role in the loss of function [9].

In contrast, neurons are not at all resistant to oxygen and glucose deprivation, ischemic conditions, and are particularly susceptible to early necrosis after insult compared to white matter. In traumatic head injury, the loss of neurons themselves (in addition to the loss in white matter conduction) plays a very substantial role in the resultant catastrophic behavioral loss, both motor/sensory, as well as cognitive. Therefore, there is relatively little time post-injury to affect a rescue of cerebral neurons of the brain when compared to the rescue of comparatively resilient white matter subsequent to SCI. Slowly degenerating white matter within the spinal cord is thus available for longer times after injury to respond to membrane – sealing agents like PEG or Poloxamer – 188. There may be other relevant factors such as changes in the vasculature with time and the sealing of the blood brain barrier at ~5 to 8 hours post-injury in the brain that may also be associated with the loss of PEG effectiveness after cerebral injury. We believe that the extended window in time for the effective use of PEG in SCI is related to these facts.

## Conclusion

These data suggest that PEG may be clinically useful to victims of TBI if delivered as rapidly as possible after injury. Presently we are beginning a veterinary clinical study of the effect of acute PEG administration in naturally injured canines suffering head injury [19]. It is possible in both dogs and humans to deliver PEG (dissolved in IV fluids) at, or close to, the accident site. Furthermore, particularly in urban areas, human patients may be admitted to emergency care within 4 hours of their accident. Still it is wise to ponder a possible precipitous decline in PEG's beneficial capabilities as an intervention in TBI and to consider ways to reduce this decline.

Since 1999, we have explored various concentrations and MWs of PEG in pursuing its use as a neuroprotective-sealing agent. Our choices in MW began with the older literature of PEG-mediated cell fusion dating to the 1970s [22,7,10] and practical concerns governing the viscosity and fluid load of injection of a *PEG solution *into animals. Importantly, the lower the molecular weight of PEG, the more toxic might be the byproducts of degradation in the body (the monomer is, of course, extremely toxic). Higher molecular weights of PEG (over 1000 daltons) pose no safety risk whatsoever and have been safely used in medicine for over 30 years [23,10]. Thus we see little room for variation from the MW and concentrations used in this series of studies. On the other hand, application via IV or another route might possibly extend the therapeutic window for use in TBI; however, we doubt this to be the case. PEG preferentially targets the damaged tissues of both spinal cord and brain while not labeling healthy or undamaged tissue [4,20]. When decorated PEG was used to determine the relative efficacy of intraveneous, intraperitoneal, or subcutaneous injection, all three types of administration similarly marked damaged spinal cord (as significantly as a topical application of PEG directly to the lesion) [20]. Thus there is little reason to believe we can improve the therapeutic benefits of PEG by changing the conventional means of administration or making different choices in the polymer's MW or concentration used for injection.

In our view, it is more likely that a combination treatment may offer the best hope of a significant clinical therapy. For example, acute administration of aldehyde scavengers [24] may prolong neuron survival in the brain long enough for PEG to exert an effect in tandem. Drugs meant to modify the deleterious effects of ischemia and reperfusion injury, unsuccessful on their own, might find renewed utility when combined with polymer infusion. We are currently considering these alternates as new plans of study.

## Abbreviations

PEG: Polytheylene Gycol; TBI: Traumatic Brain Injury; SCI: Spinal Cord Injury; CNS: Central Nervous System.

## Competing interests

Purdue University has licensed this technology to Medtronics/Sofamor Danek Corporation human clinical testing and commercialization. There is presently no financial support of this research from this corporation in support of the CPR.

## Authors' contributions

This work was in partial fulfillment of the requirements for AOK's Doctoral Degree from Purdue University. JMC worked independently on some experiments after his relocation to Dartmouth Medical School, while RBB is the Principle Investigator and Director of the TBI initiative within then CPR, and responsible for all elements of the research. All authors have read and approved the final manuscript.

## References

[B1] Vink R, O'Connor CA, Nimmo AJ, Heath DL (2003). Magnesium attenuates persistent functional deficits following diffuse traumatic brain injury in rats. Neurosci Lett.

[B2] Edwards P, Arango M, Balica L, Cottingham R, El-Sayed H, Farrell B, Fernandes J, Gogichaisvili T, Golden N, Hartzenberg B, Husain M, Ulloa MI, Jerbi Z, Khamis H, Komolafe E, Laloe V, Lomas G, Ludwig S, Mazairac G, Munoz Sanchez Mde L, Nasi L, Olldashi F, Plunkett P, Roberts I, Sandercock P, Shakur H, Soler C, Stocker R, Svoboda P, Trenkler S, Venkataramana NK, Wasserberg J, Yates D, Yutthakasemsunt S (2005). CRASH trial collaborators Final results of MRC CRASH, a randomized placebo-controlled trial of intravenous corticosteroid in adults with head injury – outcomes at 6 months. Lancet.

[B3] Lee J, Zipfel G, Choi D (1999). The changing landscape of ischaemic brain injury mechanisms. Nature.

[B4] Koob AO, Duerstock B, Babbs C, Sun Y, Borgens R (2005). Intravenous PEG inhibits loss of cerebral cells after brain injury. J Neurotrauma.

[B5] Koob AO, Borgens R (2006). Polyethylene Glycol treatment after traumatic brain injury reduces amyloid precursor protein accumulation in degenerating axons. J Neurosci Res.

[B6] Shi R, Borgens RB (2000). Anatomical repair of nerve membranes in crushed mammalian spinal cord with polyethylene glycol. J Neurocytol.

[B7] Shi R, Borgens RB, Blight AR (1999). Functional reconnection of severed mammalian spinal cord axons with polyethylene glycol. J Neurotrauma.

[B8] Luo J, Borgens RB, Shi R (2002). Polyethylene glycol immediately repairs neuronal membranes and inhibits free radical production after acute spinal cord injury. J Neurochem.

[B9] Borgens RB (2003). Restoring Function to the Injured Human Spinal Cord. Advances in Anatomy, Embryology and Cell Biology.

[B10] Borgens RB (2001). Cellular Engineering: Molecular repair of membranes to rescue cells of the damaged nervous system. Neurosurgery.

[B11] Lee J, Lentz BR (1997). Evolution of lipid structures during model membrane fusion and the relation of this process to cell membrane fusion. Biochemistry.

[B12] Kuhl T, Guo YQ, Alderfer JL, Berman AD, Leckband D, Israelachvili J, Hui SW (1996). Direct measurement of polyethylene glycol induced depletion attraction between lipid bilayers. Langmuir.

[B13] Shi R, Borgens RB (1999). Acute repair of crushed guinea pig spinal cord by polyethylene glycol. J Neurophysiol.

[B14] Luo J, Borgens RB, Shi R (2004). Polyethylene glycol improves function and reduces oxidative stress in synaptosomal preparations following spinal cord injury. J Neurotrauma.

[B15] O'Connor C, Heath DL, Cernak I, Nimmo AJ, Vink R (2003). Effects of daily versus weekly testing and pre-training on the assessment of neurologic impairment following diffuse traumatic brain injury in rats. J Neurotrauma.

[B16] Foda MA, Marmarou A (1994). A new model of diffuse brain injury in rats. Part ii: Morphological characterization. J Neurosurg.

[B17] Marmarou A, Foda MA, Brink W Van Den (1994). A new model of diffuse brain in jury in rats. Part i: Pathophysiology and biomechanics. J Neurosurgery.

[B18] Koob AO, Cirillo J, Babbs C (2006). A novel open field activity detector to determine spatial and temporal movement of laboratory animals after injury and disease. J Neurosci Methods.

[B19] Laverty P, Leskovar A, Breur G, Coates J, Bergman R, Widmer W, Toombs J, Shapiro S, Borgens R (2004). A preliminary study of intravenous surfactants in paraplegic dogs: Polymer therapy in canine clinical SCI. J Neurotrauma.

[B20] Borgens RB, Bohnert DM (2001). Rapid recovery from spinal cord injury after subcutaneously administered polyethylene glycol. J Neurosci Res.

[B21] Peasley MA, Shi R (2002). Resistance of isolated mammalian spinal cord white matter to oxygen-glucose deprivation. Am J Physiol Cell Physiol.

[B22] Borgens RB, Shi R (2000). Immediate recovery from spinal cord injury through molecular repair of nerve membranes with polyethylene glycol. FASEB J.

[B23] Working P, Newman M, Johnson J, Harris JM, Zalipsky S (1997). Safety of polyethylene glycol and polyethylene glycol derivatives, in: Polyethylene glycol Chemistry and Biological Applications.

[B24] Liu-Snyder P, Borgens R, Shi R (2006). Hydralazine Rescues PC12 Cells from Acrolein-Mediated Death. J Neurosci Res.

